# Concomitant ablation of atrial fibrillation in octogenarians: an observational study

**DOI:** 10.1186/1749-8090-3-21

**Published:** 2008-04-29

**Authors:** Herko Grubitzsch, Sven Beholz, Pascal M Dohmen, Simon Dushe, Wolfgang Konertz

**Affiliations:** 1 Department of Cardiovascular Surgery, Charité – Universitätsmedizin Berlin, Campus Charité Mitte, Berlin, Germany

## Abstract

**Background:**

Cardiac surgery is increasingly required in octogenarians. These patients frequently present atrial fibrillation (AF), a significant factor for stroke and premature death. During the last decade, AF ablation has become an effective procedure in cardiac surgery. Because the results of concomitant AF ablation in octogenarians undergoing cardiac surgery are still not clear, we evaluated the outcome in these patients.

**Methods:**

Among 200 patients undergoing concomitant AF ablation (87% persistent AF), 28 patients were ≥ 80 years (82 ± 2.4 years). The outcome was analysed by prospective follow up after 3, 6, 12 months and annually thereafter. Freedom from AF was calculated according to the Kaplan-Meier method.

**Results:**

Octogenarians were similar to controls regarding AF duration (48 ± 63.2 versus 63 ± 86.3 months, n.s.) and left atrial diameter (49 ± 6.1 versus 49 ± 8.8 mm, n.s.), but differed in EuroSCORE (17.3 ± 10.93 versus 7.4 ± 7.31%, p < 0.001), prevalence of paroxysmal AF (25.0 versus 11.0%, p = 0.042) and aortic valve disease (67.8 versus 28.5%, p < 0.001). ICU stay (8 ± 16.9 versus 4 ± 7.2 days, p = 0.027), hospital stay (20 ± 23.9 versus 14 ± 30.8 days, p < 0.05), and 30-d-mortality (14.3 versus 4.6%, p = 0.046) were increased. After 12 ± 6.1 months of follow-up (95% complete), 14 octogenarians (82%) and 101 controls (68%, n.s.) were in sinus rhythm; 59% without antiarrhythmic drugs in either group (n.s.). Sinus rhythm restoration was associated with improved NYHA functional class and renormalization of left atrial size. Cumulative freedom from AF demonstrated no difference between groups. Late mortality was higher in octogenarians (16.7 versus 6.1%, p = 0.065).

**Conclusion:**

Sinus rhythm restoration rate and functional improvement are satisfactory in octogenarians undergoing concomitant AF ablation. Hence, despite an increased perioperative risk, this procedure should be considered even in advanced age.

## Background

Because industrialized countries are experiencing an aging population, the age of patients necessitating cardiac surgery is steadily rising. According to the registry of the German Society for Thoracic and Cardiovascular Surgery, more than 8.4% of patients undergoing cardiac surgical procedures in 2005 were older than 80 years, compared to 2.7% ten years ago [1]. It was shown that these high risk elderly patients derive benefit from coronary revascularization, aortic as well as mitral valve surgery, and combined procedures [2-4].

Atrial fibrillation (AF), a significant factor for stroke and premature death, is the most frequent sustained atrial arrhythmia [5]. Because its incidence increases with age, a growing number of patients scheduled for valvular or coronary heart surgery, nowadays present AF [6,7]. During the last decade, surgical AF treatment, initially introduced as Maze procedure [8,9], has been developed to a less complex operation by using different technologies for tissue ablation and by focussing the lesion pattern on the left atrium [7,10,11]. AF ablation has become a frequently performed concomitant procedure in cardiac surgery with overall promising results regarding sinus rhythm (SR) restoration [7,10].

Because the results of surgical AF treatment in octogenarians are not clear, we evaluated outcome of these patients undergoing cardiac surgery and concomitant AF ablation.

## Methods

### Patients

Between January 2003 and February 2006, a total of 200 patients (54.5% male) scheduled for cardiac surgery underwent concomitant AF ablation. The majority of patients presented with persistent/continuous AF (n = 174, 87.0%) according to established definitions [12,13]. Out of all, 28 patients aged 80 years and older were summarized as group of octogenarians. According to age (< 80 years versus ≥ 80 years), patients were prospectively studied. Preoperative, perioperative and follow up data were entered into an institutional database. Informed consent regarding the operation and data acquisition was obtained from all patients. For assessment of perioperative risk, logistic EuroSCORE was determined [14].

### Surgical procedures and perioperative treatment

The detailed procedures of the study population are listed in Table 1. For all procedures, standard normothermic cardiopulmonary bypass and warm antegrade blood cardioplegia were used. All patients underwent endocardial ablation of the left atrium as described in detail previously [7]. The left atrial appendage was oversewn only if thrombi were inside (n = 4).

**Table 1:** Surgical procedures

	Overall (n = 200) % (n)	Age < 80 yrs (n = 172) % (n)	Age ≥ 80 yrs (n = 28) % (n)
MVP/-R	37.5 (75)	41.3 (71)	14.3 (4)
isolated	57.3 (43)	59.2 (42)	25.0 (1)
+ CABG	28.0 (21)	25.4 (18)	75.0 (3)
+ TVP/R/+ CABG/+ congenital	14.7 (11)	15.5 (11)	-
AVR	34.0 (68)	28.5 (49)	67.8 (19)
isolated	58.8 (40)	61.2 (30)	52.6 (10)
+ CABG	11.8 (8)	6.1 (3)	26.3 (5)
+ MVP/-R/+ TVP/-R/+ CABG/+ AAR	29.4 (20)	32.6(16)	21.1 (4)
CABG	28.0 (56)	29.6 (51)	17.8 (5)
isolated	98.2 (55)	98.0 (50)	100 (5)
+ aneurysmectomy	1.8 (1)	2.0 (1)	-
other procedures	0.5 (1)	0.6 (1)	-
LA reduction plasty	1.5 (3)	1.7 (3)	-
reoperation	7.0 (14)	7.6 (13)	3.6 (1)
microwave ablation	53.5 (107)	52.9 (91)	57.1 (16)
radiofrequency ablation	46.5 (93)	47.1 (81)	42.8 (12)

AAR, ascending aortic replacement, AVR, aortic valve replacement; CABG, coronary artery bypass grafting; MVP/-R, mitral valve plasty/replacement; TVP/R, tricuspid valve plasty/replacement; yrs, years

All patients were anticoagulated with heparin followed by phenprocoumon with a target INR of 2.0–3.0. After 3 months and stable sinus or atrial driven pacemaker rhythm in Holter ECG and mechanical atrial function in echocardiogram anticoagulation was ceased. Patients with mechanical valve substitutes were kept on phenprocoumon permanently (INR of 2.5–3.5 for aortic and 3.0–4.0 for mitral valve prostheses). DC shock cardioversion of early recurrent AF was performed if patient was symptomatic or hemodynamically compromised. Perioperatively, either patient's preoperative betablocker was continued or antiarrhythmic treatment with class III antiarrhythmic drugs (sotalol or amiodarone) was initiated. The decision was left to the discretion of the surgeon. After discharge, patient's general physician or cardiologist managed the anticoagulation and antiarrhythmic therapy.

### Follow up

Prospective follow up (FU) was performed after 3, 6, 12 months and annually thereafter. Patients were interviewed and underwent clinical examination, electrocardiography, and transthoracic echocardiography (TTE). Only in 9 patients (4.8%), who were not able to visit the clinic, interviewing was done by telephone and echocardiographic data were obtained from the referring cardiologist. Ablation was considered successful if SR was maintained with no symptomatic or documented episodes of AF or atrial flutter. Any regular atrial driven rhythm, including atrial (n = 1), atrioventricular (n = 3), or atrial triggered ventricular (n = 5) pacing, was regarded as sinus rhythm (SR).

### Echocardiography

Preoperatively, before discharge and at FU, all patients underwent TTE using the HP Sonos 5500 (Hewlett Packard, Andover, Massachusetts, USA). LA and LV diameter were measured using standard techniques. LVEF was assessed by the Simpson method. For assessment of LA function the pulsed-wave signal of diastolic transmitral flow was used. Maximal flow velocities of E and A waves were measured and E/A ratio was calculated.

### Statistical analysis

Unless otherwise indicated, data are presented as mean ± standard deviation or absolute and relative frequencies. For comparison between groups, Mann-Whitney's U-test (continuous variables) and Fisher's exact test (categorical variables) were used. For comparison of FU and preoperative data within groups Wilcoxon's rank-sum test was applied. All tests of significance were two-tailed and a value of p < 0.05 was considered significant. During FU, freedom from AF was calculated according to the Kaplan-Meier method. Differences were analysed by log-rank test. Statistical analysis was performed using a statistical software program (SPSS 13.0 for Windows, SPSS Inc., Chicago, Illinois, USA).

## Results

### Patient groups

The mean age of octogenarians was 82 ± 2.4 years (range 80–89 years), whereas the mean age of younger patients was significantly lower at 68 ± 7.9 years (range 40–79 years). Baseline characteristics are presented in Table 2. There was a higher proportion of women in octogenarians which was associated with a lower body surface area. The pattern of valvular heart disease was significantly different in both groups with respectively higher incidence of mitral valve disease in younger and aortic valve disease in older patients. There was a non-significant trend to an increased percentage of patients with a history of thrombembolic events in octogenarians. AF duration and LA size, two important AF criteria, were similar in either group, although a higher proportion of younger patients were in persistent AF. According to New York Heart Association (NYHA) functional class and EuroSCORE, older patients were significantly more compromised and exhibited a higher perioperative risk.

**Table 2:** Baseline data

		Age < 80 yrs (n = 172)	Age ≥ 80 yrs (n = 28)	p
male gender	% (n)	57.6 (99)	35.7 (10)	0.031
body surface area	m^2^	1.9 ± 0.22	1.8 ± 0.18	0.003
coronary artery disease	% (n)	45.3 (78)	50.0 (14)	0.647
mitral valve disease	% (n)	50.0 (86)	28.6 (8)	0.035
aortic valve disease	% (n)	28.5 (49)	67.8 (19)	< 0.001
others	% (n)	9.3 (16)	-	-
previous embolism	% (n)	1.7 (3)	7.1 (2)	0.089
persistent AF	% (n)	89.0 (153)	75.0 (21)	0.042
AF duration	months	63 ± 86.3	48 ± 63.2	0.400
LA diameter	mm	49 ± 8.8	49 ± 6.1	0.954
LVEF		0.51 ± 0.013	0.52 ± 0.014	0.656
LVEDD	mm	54 ± 8.1	49 ± 10.4	0.379
NYHA class		2.9 ± 0.81	3.4 ± 0.67	0.021
logistic EuroSCORE	%	7.4 ± 7.31	17.3 ± 10.93	< 0.001

AF, atrial fibrillation; LA, left atrial; LVEDD, left ventricular enddiastolic diameter; LVEF, left ventricular ejection fraction; NYHA, New York Heart Association; yrs, years

### Procedural outcome

In octogenarians and younger patients, there was no difference in aortic cross clamp time (90 ± 29.2 and 85 ± 32.0 min, p = 0.464), cardiopulmonary bypass time (125 ± 31.9 and 116 ± 38.5 min, p = 0.568), and operation time (206 ± 41.0 and 217 ± 60.0 min, p = 0.389). Post ablation treatment with antiarrhythmic drugs or betablockers was similar in both groups (Table 3). In 6 patients, a dual chamber pacemaker was implanted due to sinus node dysfunction. Regarding perioperative morbidity, the incidence of heart failure requiring intraaortic balloon pump support and the incidence of renal failure was significantly higher in octogenarians. All patients of this group who acquired infections early after operation developed sepsis. The incidence of cerebral ischemic events was similar in younger and older patients. Overall, 2 strokes (on postoperative day [POD] 1 and 5), and 2 transient neurological deficits (on POD 2 and 6) occurred. Multiple thrombembolism due to hereditary thrombotic thrombocytopenic purpura occurred in one of these patients on POD 5. Apart from a trend to prolonged mechanical ventilation, older age was associated with significantly longer ICU and hospital stay. Perioperative mortality was higher in octogenarians. No death was ablation related.

**Table 3:** Perioperative data

		Age < 80 yrs (n = 172)	Age ≥ 80 yrs (n = 28)	p
betablocker	% (n)	27.9 (48)	17.8 (5)	0.172
class III antiarrhythmic drugs	% (n)	52.3 (90)	60.7 (17)	0.409
Sotalol	% (n)	76.7 (69)	58.8 (10)	0.125
Amiodaron	% (n)	23.3 (21)	41.2 (7)	0.125
DC shock cardioversion	% (n)	4.6 (8)	3.6 (1)	0.798
pacemaker implantation	% (n)	3.5 (6)	-	-
re-exploration for bleeding	% (n)	2.3 (4)	-	-
pericardial effusion	% (n)	1.2 (2)	-	-
heart failure	% (n)	4.1 (7)	7.1 (2)	0.467
inotropic support	% (n)	4.1 (7)	7.1 (2)	0.467
IABP	% (n)	2.9 (5)	5.1 (4)	0.045
LVAD	% (n)	1.2 (2)	-	-
pulmonary failure	% (n)	-	3.6 (1)	-
renal failure	% (n)	7.0 (12)	17.8 (5)	0.056
cerebrovascular accident	% (n)	1.7 (3)	3.6 (1)	0.522
intracerebral hemorrhage	% (n)	0.6 (1)	-	-
infection	% (n)	4.6 (8)	10.7 (3)	0.192
mediastinitis	% (n)	0.6 (1)	3.6 (1)	0.140
bronchopulmonary infection	% (n)	4.1 (7)	7.1 (2)	0.467
sepsis	% (n)	2.3 (4)	10.7 (3)	0.025
mechanical ventilation time	hrs	21 ± 50.3	28 ± 41.1	0.069
ICU stay	days	4 ± 7.2	8 ± 16.9	0.027
hospital stay	days	14 ± 30.8	20 ± 23.9	0.004
mortality (30 days)	% (n)	4.6 (8)	14.3 (4)	0.046
cardiac death	% (n)	0.6 (1)	3.6 (1)	0.140
non-cardiac death	% (n)	4.1 (7)	10.7 (3)	0.135

DC, direct current; IABP, intraaortic balloon pump; ICU, intensive care unit; LVAD, left ventricular assist device; yrs, years

### Outcome during follow up

**Figure 1:**
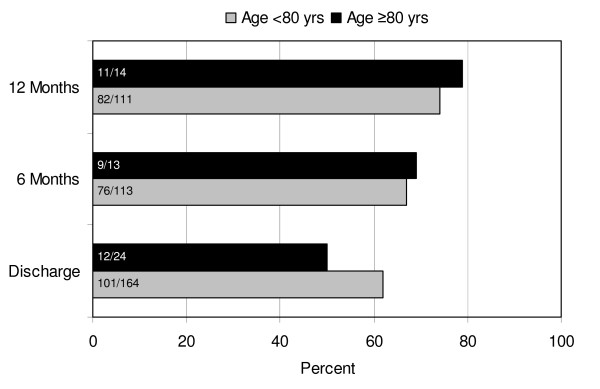
**Sinus rhythm at follow up**. Figures in bars indicate absolute frequencies of patients in SR.

**Figure 2:**
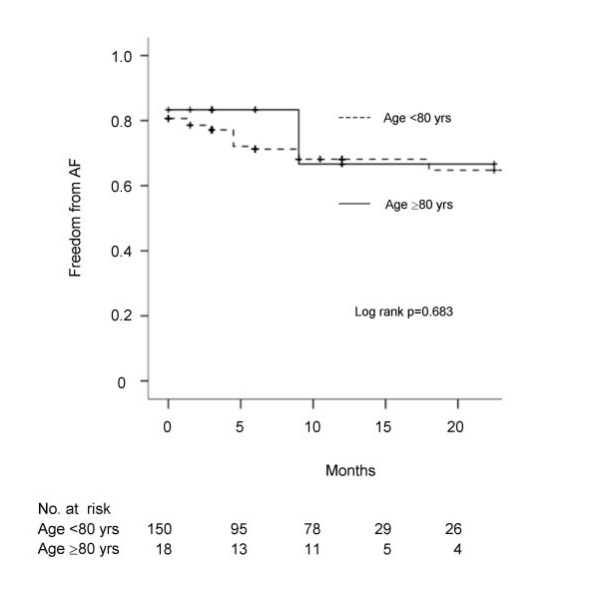
**Kaplan-Meier estimates**. Freedom from persistent AF in patients </≥ 80 years. AF, atrial fibrillation; yrs, years.

Mean FU after 12 ± 6.1 months was 95% complete; 9 patients were lost. During FU, mortality was higher in octogenarians (16.7% versus 6.1%, p = 0.065). Causes of late death were cardiac in 3 and 1, non-cardiac in 6 and 2, and unknown in either 1 younger and older patients, respectively. In neither group, thrombembolic complications occurred. Compared to discharge, there was a trend to higher SR conversion rate after 6 and 12 months (Figure 1). Neither between cohorts, nor at different FU visits, statistical significance was reached. Results regarding rhythm outcome are outlined in Table 4. At latest available FU, 14 octogenarians (82%) and 101 younger patients (68%, p = 0.231) were in sinus rhythm (SR); 59% without antiarrhythmic drugs in either group (p = 0.997). Recurrent tachyarrhythmia was successfully treated by DC shock cardioversion in 3 patients aged < 80 years and 1 patient ≥ 80 years and by interventional ablation in 2 younger patients. The majority of patients in SR presented normal atrial contraction (Table 4). Compared to patients who were not in SR, patients with restored SR at FU presented smaller LA size (41 ± 5.3 versus 45 ± 7.3 mm, p = 0.005 and 41 ± 8.8 versus 46 ± 11.6 mm, p = 0.498) and better NYHA functional class (1.8 ± 0.60 versus 2.2 ± 0.56, p < 0.001 and 1.9 ± 0.48 versus 2.3 ± 0.58, p = 0.203) in younger and older age group, respectively. According to LVEF, left ventricular function remained unchanged in either group. Kaplan-Meier estimates of freedom from persistent AF demonstrated no difference between both groups (Figure 2).

**Table 4:** Outcome at last follow up

		Age < 80 yrs (n = 148)	Age ≥ 80 yrs (n = 17)	p
sinus rhythm	% (n)	68.2 (101)	82.3 (14)	0.231
atrial contraction	% (n)	87.8 (79)	100 (12)	0.200
E/A ratio		2.5 ± 0.83	2.1 ± 0.75	0.179
atrial fibrillation	% (n)	26.3 (39)	11.8 (2)	0.187
atrial flutter	% (n)	3.4 (5)	5.9 (1)	0.601
NYHA class		1.9 ± 0.61*	2.1 ± 0.57*	0.304
LA diameter	mm	42 ± 6.3*	42 ± 9.1*	0.952
LVEF		0.52 ± 0.120^†^	0.55 ± 0.091*	0.358
LVEDD	mm	54 ± 9.4^†^	49 ± 8.3^†^	0.048

*p < 0.001, comparison to preoperative data by Wilcoxon rank-sum test

^†^p = n.s., comparison to preoperative data by Wilcoxon rank-sum test

E/A ratio, ratio of maximal flow velocities of early (E) and atrial (A) diastolic transmitral flow signal assessed by pulsed-wave Doppler; NYHA, New York Heart Association; LA, left atrial; LVEF, left ventricular ejection fraction; LVEDD, left ventricular enddiastolic diameter

Assessment of LA function by pulsed wave Doppler failed in 11 and 2 patients in the younger and older age group, respectively.

## Discussion

A constant extension of life expectancy in industrialized countries together with an increased incidence of cardiovascular disease is leading to a rising number of elderly patients referred for cardiac surgery. These patients frequently present with AF, because the prevalence of this arrhythmia is exponentially age-related [6]. Whereas AF occurs in less than 0.5% of patients younger than 60 years, approximately 10% of patients older than 80 years are in AF [6]. Because promising results have been reported on valvular and coronary surgery in octogenarians [2-4], we evaluated the outcome of patients in this age group after concomitant AF ablation.

In this study, octogenarians were different from younger controls in that they were more likely to be female and require aortic valve replacement. Either distribution refers to demographic characteristics and is consistent with previous reports [15,16]. On the other hand, the underlying heart disease does not determine results of AF ablation [7]. The observed inferior functional status according to NYHA class and the trend to higher incidence of preoperative thrombembolic events may reflect general findings in advanced age [2]. As age is one of the most important predictors for operative mortality [14], patients ≥ 80 years exhibited a significantly higher risk reflected by EuroSCORE. According to recent reports, early mortality after cardiac surgery in octogenarians ranges from 7.5% to 26.5% (table 5). Apart from age, NYHA functional class [3,17,18], presence of heart failure as well as left ventricular dysfunction [3,15], pulmonary hypertension [17], and renal insufficiency [3,19] were demonstrated to be predictive factors for mortality. Furthermore, procedural factors as urgent/emergency and re-do surgery [3,4,18-20] or combined valvular plus revascularization surgery [4] increase operative risk. In this study, patients ≥ 80 years who died early after operation were characterized by LVEF ≤ 0.40 (n = 2), re-do surgery (n = 1), and other than isolated CABG (n = 4) resulting in a mean logistic EuroSCORE of 25.6%. Interestingly, causes of death were mainly non-cardiac. As reported very recently, AF per se seems to be a marker for higher operative risk [21,22]. However, the particular impact of AF ablation on perioperative mortality has not been clarified, yet. Analysis of overall complications early after operation (table 3) reflects the fragility of octogenarians due to age-related alterations in different organ systems. As in our patients for instance, renal failure frequently occurs after cardiac surgery in octogenarians [2,19] and contributes to operative risk [20]. In contrast to others [2,19,20], we did not observe an increase in postoperative stroke in our small cohort of patients. With respect to mechanical ventilation time, length of ICU and hospital stay, and incidence of postoperative complications, increased resource utilization has to be considered in octogenarians undergoing cardiac surgery [2].

**Table 5:** Outcome of octogenarians undergoing cardiac surgery

	procedure	author	year	n	operative mortality	longterm survival	
						1 year	5 years
CABG	isolated	Alexander [19]	2000	4306	8.1% (hospital)	-	-
	isolated	Kolh [18]	2001	70	10.0% (hospital)	-	65.8%
	isolated	Scott [2]	2005	155	9.0% (30 days)	-	-
							
AVR	combined	Alexander [19]	2000	345	10.1% (hospital)	-	-
	isolated and combined	Sundt [20]	2000	133	11.0% (30 days)	80.0%	55.0%
	isolated	Kolh [18]	2001	70	8.5% (hospital)	-	63.6%
	combined	Kolh [18]	2001	30	26.5% (hospital)	-	62.4%
	isolated and combined	Chiappini [23]	2004	115	8.5% (hospital)	86.4%	69.4%
	isolated and combined	Langanay [3]	2006	442	7.5%	-	-
							
MVP-/R	combined	Alexander [19]	2000	92	19.7% (hospital)	-	-
	isolated	Kolh [18]	2001	12	25.0% (hospital)		57.1%
	isolated and combined	Nagendran [4]	2005	58	15.5% (hospital)	-	70.0%

AVR, aortic valve replacement; CABG, coronary artery bypass grafting; MVP-/R, mitral valve plasty/replacement

Overall survival of 82% after mean FU of 12 months was comparable with outcome after isolated or combined aortic valve replacement (table 5) [20,23]. As AF prevalence is high in patients older than 80 years [6], it seems obvious that advanced age predicted recurrent AF after the Cox Maze procedure in mitral valve disease [24]. Nonetheless, we found, that SR can be restored in the majority of elderly patients leading to normal atrial contraction and symptomatic improvement regarding NYHA functional class. Although octogenarians more frequently presented with paroxysmal AF at baseline, 71.4% of patients ≥ 80 years with persistent AF before operation were in SR at last FU. Moreover, freedom from AF during FU was not different in octogenarians and younger patients (figure 2). The overall SR conversion rate observed in this study is within the range of 57–92%, reported in a recent metaanalysis [25]. At least in part, this could be attributed to the left atrial in contrast to the classical biatrial approach. However, a less complex procedure for concomitant AF treatment might be beneficial, in particular for octogenarians. Furthermore, it was shown that AF will not recur if macro-reentry can be prevented by lesions critically placed in the left atrium [11,13].

## Conclusion

This is the first report on concomitant AF ablation in octogenarians undergoing cardiac surgery. The main finding is, that SR and atrial transport function could be restored in the majority of patients. Certainly, ongoing research has to prove these results in larger cohorts and during long-term follow up. Because this study is a retrospective analysis of prospectively collected data it suffers from general limitations inherent to observational studies in non-randomized patient groups. Hence, we are unable to generalize our results to all patients ≥ 80 years with AF necessitating cardiac surgery. Preoperative identification of candidates in whom concomitant AF ablation will improve outcome is indeed challenging, especially if perioperative risk and a certain percentage of patients remaining in AF are considered. Nevertheless, our finding suggests integration of concomitant AF ablation as reasonable treatment strategy in elderly patients with AF referred for cardiac surgery.

## List of abbreviations used

AAR: ascending aortic replacement; AF: atrial fibrillation; AVR: aortic valve replacement; CABG: coronary artery bypass grafting; DC: direct current; ECG: electrocardiogram; FU: follow up; IABP: intraaortic balloon pump; ICU: intensive care unit; INR: international normalized ratio; LA: left atrial; LV: left ventricular; LVAD: left ventricular assist device; LVEDD: left ventricular enddiastolic diameter; LVEF: left ventricular ejection fraction; MVP/-R: mitral valve plasty/replacement; n.s., not significant; NYHA: New York Heart Association; POD: postoperative day; SR: sinus rhythm; TTE: transthoracic echocardiography; TVP/R: tricuspid valve plasty/replacement; Yrs, years

## Competing interests

The authors declare that they have no competing interests.

## Authors' contributions

HG designed the study, performed statistical analysis and drafted the manuscript. SB helped to design the study and participated in data acquisition. SD performed echocardiographic examinations. PMD participated in data acquisition. WK participated in study coordination and revised the manuscript. All authors read and approved the final version.
